# Crystal structure of 1-(2-fluoro­benzo­yl)-2,7-di­meth­oxy­naphthalene

**DOI:** 10.1107/S1600536814020807

**Published:** 2014-10-04

**Authors:** Saki Mohri, Shinji Ohisa, Takehiro Tsumuki, Noriyuki Yonezawa, Akiko Okamoto

**Affiliations:** a Department of Organic and Polymer Materials Chemistry, Tokyo University of Agriculture & Technology (TUAT), Koganei, Tokyo 184-8588 , Japan

**Keywords:** crystal structure, 1-aroyl­naphthalene compound, non-coplanarly accumulated aromatic rings structure, spatial organization

## Abstract

The asymmetric unit of the compound, C_19_H_15_FO_3_, contains two independent molecules. Each molecule has essentially the same feature of non-coplanarly accumulated aromatic rings whereby the aroyl group at the 1-position of the naphthalene ring system is twisted almost perpendicularly to the ring system [dihedral angles of 86.52 (8) and 89.66 (8)°]. In the crystal structure, mol­ecules of the same conformer are stacked into columns parallel to the *a* axis by van der Waals inter­actions only.

## Abstract 2

The asymmetric unit of the compound contains two independent conformers. Each conformer is stacked along the *a* axis to form columns through van der Waals inter­actions only.

## Chemical context   

Compounds with non-coplanarly accumulated aromatic rings have received attention as unique structural building blocks from organic chemists and materials chemists, because they provide characteristic optical and electronic properties originating from their structural features. For example, biphenyl and binaphthyl are applied to optically active mol­ecular catalysts and polymer materials on the basis of their axial chiralities (Pravas *et al.*, 2013 ▶). In the course of our study on selective electrophilic aromatic aroylation of 2,7-di­meth­oxy­naph­thalene, it was found that *peri*-aroyl­naphthalene compounds are formed regioselectively with the aid of suitable acidic mediators (Okamoto & Yonezawa, 2009 ▶; Okamoto *et al.*, 2012 ▶). The X-ray analyses of *peri*-aroyl­naphthalene compounds revealed that the aroyl groups at the 1- and 8-positions of the naphthalene ring systems are connected almost perpendicularly but the benzene rings of the aroyl groups tilt slightly toward the *exo* sides of the naphthalene ring systems, as observed in 1,8-dibenzoyl-2,7-di­meth­oxy­naphthalene (Nakaema *et al.*, 2008 ▶) and (2,7-di­meth­oxy­naphthalene-1,8-di­yl)bis­(4-fluoro­benzo­yl)di­meth­an­one (Wat­anabe *et al.*, 2010 ▶). Moreover, the homologous 1-(4-substituted benzo­yl)naphthalenes also have essentially the same non-coplanar structure of the corresponding 1,8-diaroylated naphthalenes, *e.g*. (2,7-di­meth­oxy­naphthalen-1-yl)(phen­yl)methanone (Kato *et al.*, 2010 ▶) and (2,7-di­meth­oxy­naphthalen-1-yl)(4-fluoro­phen­yl)methanone (Watan­abe *et al.*, 2011 ▶). On the other hand, dynamic NMR study has clarified the difference between 1-benzoyl­ated and 1,8-di­benzoyl­ated naphthalene (Okamoto *et al.*, 2011 ▶). In solution, the carbon–carbon bond rotation involving the benzoyl group and the naphthalene ring system in 1,8-dibenzoyl-2,7-di­meth­oxy­naphthalene is rather restricted, whereas the spatial organization of 1-benzoyl-2,7-di­meth­oxy­naphthalene changes flexibly through the bond rotation. As part of our study on the mol­ecular structures of this kind of homologous mol­ecules, the crystal structure of title compound, a 1-benzoyl­ated naphthalene bearing the fluoro group at the 2-position of the benzoyl moiety, is discussed in this paper.
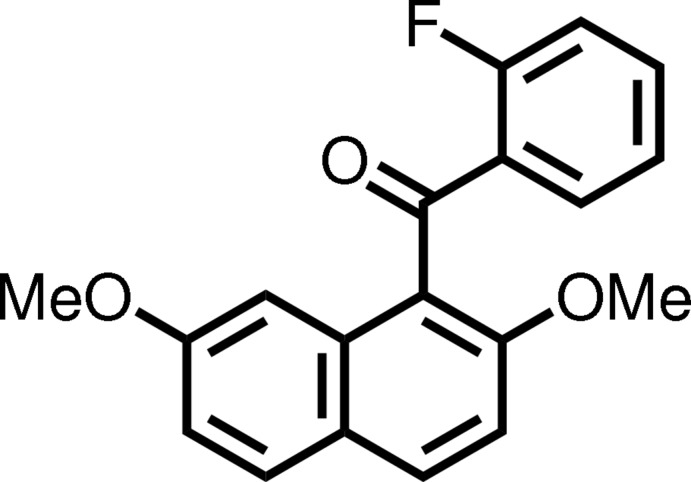


## Structural commentary   

**Figure 1:**
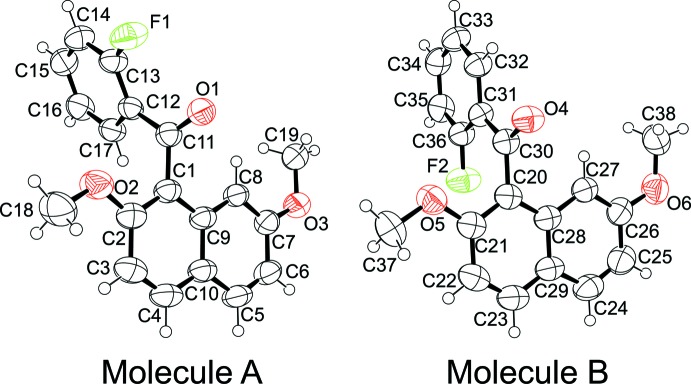
The mol­ecular structure of the two conformers of the title compound, with displacement ellipsoids drawn at the 50% probability level.

There are two independent conformers in the asymmetric unit of the title compound. The independent conformers (*A* and *B*) are shown in Fig. 1 ▶. Each conformer has essentially the same non-coplanar structure. However, the orientation of the 2-fluoro­phenyl group against the naphthalene ring system is different in conformer (*A*) and (*B*), *i.e.*, *exo*-side for conformer (*A*) and *endo*-side for conformer (*B*). The dihedral angle between the naphthalene ring system and the benzene ring of the 2-fluoro­benzoyl group is 86.52 (8) for conformer *A* and 89.66 (8)° for *B*. Bond distances and angles are not unusual.

## Supra­molecular features   

**Figure 2:**
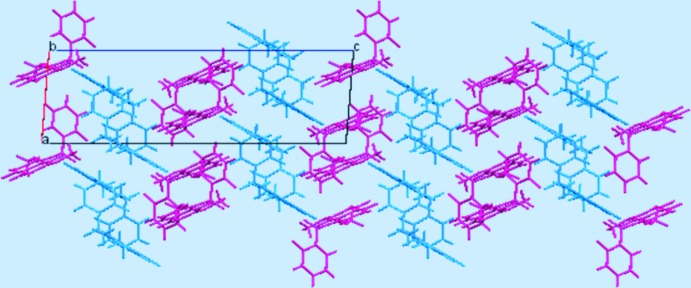
Crystal packing of the title compound viewed along the *b* axis. Conformers *A* and *B* are drawn in purple and blue, respectively.

In the crystal structure, mol­ecules of the same conformer are stacked along the *a* axis through weak van der Waals inter­actions into a columnar array (Fig. 2 ▶). No hydrogen bonds or π–π stacking inter­actions are observed. Intra- and inter­columnar C—H⋯π contacts with an H⋯π(centroid) separation slightly shorter than 3 Å are present (H32⋯*Cg*1 = 2.97; H16⋯*Cg*2^i^ = 2.94; H35⋯*Cg*3^i^ = 2.90 Å; *Cg*1, *Cg*2 and *Cg*3 are the centroids of the C12–C17, C1–C6, and C24–C29 rings, respectively; symmetry code: (i) 1-*x*, −*y*, −*z*), but their significance as structure-directing inter­actions is doubtful.

## Database survey   

A search of the Cambridge Structural Database (Version 5.35, last update May 2014; Allen, 2002 ▶) showed 19 and 12 structures containing the 1-substituted-2,7-di­alk­oxy­naphthalene (including 1-acetyl­naphthalene) and 1-aroyl-2,7-di­alk­oxy­naphthalene units, respectively. The title compound has a non-coplanarly accumulated aromatic ring structure, as found in the fluoro-group-free 1-benzoyl­naphthalene homologues and the fluoro-group-bearing 1-benzoyl­naphthalene homologue, *viz*. 1-benzoyl-2,7-di­meth­oxy­naphthalene (Kato *et al.*, 2010 ▶) and 1-(4-fluoro­benzo­yl)-2,7-di­meth­oxy­naphthalene (Watanabe *et al.*, 2011 ▶). Both homologues form a columnar structure *via* C–H⋯O=C hydrogen bonds. In the case of the fluoro-group-free homologue, three conformers are found, each of them forming a columnar structure *via* C–H⋯O=C hydrogen bonds. The title compound forms a columnar structure similar to the homologues without C—H⋯O=C interactions in the crystal. Therefore, 1-benzoylnaphthalene homologues might be susceptible to form the columnar structure. The C—H⋯O=C hydrogen bonds plausibly contribute to pack the molecules densely within the column, as indicated by the densities of the title compound (1342 Mg m^−3^) and the 4-fluorobenzoyl group-bearing homologue (1.351 Mg m^−3^). However, the number of conformers seems to afford a larger influence on the whole of the crystal packing. When several types of conformer are formed, intracolumnar interactions should be enhanced. In other words, intercolumnar interactions relatively weaken compared with the intracolumnar interactions. Consequently, the densities are apparently different between the title compound and the fluoro-group-free homologue (1.276 Mg m^−3^).

## Synthesis and crystallization   

To a test-tube-type flask, 2-fluoro­benzoyl chloride (1.1 mmol, 0.130 ml), aluminium chloride (AlCl_3_; 1.3 mmo1, 0.173 g), and methyl­enechloride (CH_2_Cl_2_; 2.0 ml) were placed and stirred at 273 K. To the reaction mixture thus obtained 2,7-di­meth­oxy­naphthalene (1.0 mmol, 0.188 g) was added. After the reaction mixture had been stirred at 273 K for 4 h, it was poured into methanol (10 ml) and water (20 ml) and the mixture was extracted with CHCl_3_ (10 ml × 3). The combined extracts were washed with aqueous 2*M* NaOH followed by washing with brine. The organic layers obtained were dried over anhydrous MgSO_4_. The solvent was removed under reduced pressure to give a cake. The crude product was purified by recrystallization from hexane (isolated yield 63%). Single crystals suitable for X-ray analysis were obtained from the isolated product by slow evaporation of a CHCl_3_/hexane (1:3 *v*/*v*) solution.

^1^H NMR δ (300 MHz, CDCl_3_): 3.75 (3H, *s*), 3.78 (3H, *s*), 7.07 (4H, *m*), 7.19 (1H, *t*, *J* = 7.6 Hz), 7.51 (1H, *m*), 7.74 (2H, *m*), 7.86 (1H, *d*, *J* = 8.7 Hz) p.p.m. ^13^C NMR δ (75 MHz, CDCl_3_): 31.19, 31.23, 53.95, 55.30, 56.47, 60.94, 76.71, 77.13, 77.55, 102.05, 110.37, 116.84, 117.25, 124.19, 124.24, 124.60, 129.83, 131.49, 131.87, 134.41, 155.94, 159.30, 159.97, 163.40, 194.56 p.p.m. IR (KBr): 1668 (C=O), 1605, 1511, 1479 (Ar, naphthalene), 1233 (=C—O—C) cm^−1^. HRMS (*m/z*): [*M* + H]^+^ Calculated for C_19_H_15_FO_3_, 310.1042; found, 310.1005; m.p. = 365.2–365.7 K.

## Refinement   

Crystal data, data collection and structure refinement details are summarized in Table 1 ▶. All H atoms were located in a difference Fourier map and were subsequently refined as riding atoms, with C—H = 0.95–0.98 Å, and with *U*_iso_(H) = 1.2 *U*_eq_(C). The positions of methyl H atoms were rotationally optimized.

## Supplementary Material

Crystal structure: contains datablock(s) I. DOI: 10.1107/S1600536814020807/rz5132sup1.cif

Structure factors: contains datablock(s) I. DOI: 10.1107/S1600536814020807/rz5132Isup2.hkl

Supporting information file. DOI: 10.1107/S1600536814020807/rz5132Isup3.pdf

Supporting information file. DOI: 10.1107/S1600536814020807/rz5132Isup4.pdf

Supporting information file. DOI: 10.1107/S1600536814020807/rz5132Isup5.pdf

Supporting information file. DOI: 10.1107/S1600536814020807/rz5132Isup6.pdf

Supporting information file. DOI: 10.1107/S1600536814020807/rz5132Isup7.cml

CCDC reference: 1024598

Additional supporting information:  crystallographic information; 3D view; checkCIF report

## Figures and Tables

**Table 1:** Experimental details

Crystal data	
Chemical formula	C_19_H_15_FO_3_
*M*_r_	310.31
Crystal system, space group	Monoclinic, *P*2_1_/*n*
Temperature (K)	193
*a*, *b*, *c* ()	8.36074(15), 15.5479(3), 23.6898(4)
()	94.163(1)
*V* (^3^)	3071.36(10)
*Z*	8
Radiation type	Cu *K*
(mm^1^)	0.82
Crystal size (mm)	0.50 0.30 0.20

Data collection	
Diffractometer	Rigaku R-AXIS RAPID
Absorption correction	Numerical (*NUMABS*; Higashi, 1999 ▶)
*T*_min_, *T*_max_	0.686, 0.854
No. of measured, independent and observed [*I* > 2(*I*)] reflections	54661, 5600, 4226
*R*_int_	0.032
(sin /)_max_ (^1^)	0.602

Refinement	
*R*[*F* ^2^ > 2(*F* ^2^)], *wR*(*F* ^2^), *S*	0.043, 0.125, 1.04
No. of reflections	5600
No. of parameters	420
H-atom treatment	H-atom parameters constrained
_max_, _min_ (e ^3^)	0.29, 0.20

Computer programs: *PROCESS-AUTO* (Rigaku, 1998 ▶), *CrystalStructure* (Rigaku, 2010 ▶), *SIR2004* (Burla *et al.*, 2007 ▶), *SHELXL97* (Sheldrick, 2008 ▶) and *ORTEPIII* (Burnett Johnson, 1996 ▶).

## supplementary crystallographic information

### Crystal data

**Table:**

C_19_H_15_FO_3_	*F*(000) = 1296
*M**_r_* = 310.31	*D*_x_ = 1.342 Mg m^−^^3^
Monoclinic, *P*2_1_/*n*	Cu *K*α radiation, λ = 1.54187 Å
Hall symbol: -P 2yn	Cell parameters from 43860 reflections
*a* = 8.36074 (15) Å	θ = 3.4–68.2°
*b* = 15.5479 (3) Å	µ = 0.82 mm^−^^1^
*c* = 23.6898 (4) Å	*T* = 193 K
β = 94.163 (1)°	Block, colorless
*V* = 3071.36 (10) Å^3^	0.50 × 0.30 × 0.20 mm
*Z* = 8	

### Data collection

**Table:**

Rigaku R-AXIS RAPID diffractometer	5600 independent reflections
Radiation source: fine-focus sealed tube	4226 reflections with *I* > 2σ(*I*)
Graphite monochromator	*R*_int_ = 0.032
Detector resolution: 10.000 pixels mm^-1^	θ_max_ = 68.2°, θ_min_ = 3.4°
ω scans	*h* = −9→9
Absorption correction: numerical (*NUMABS*; Higashi, 1999)	*k* = −18→18
*T*_min_ = 0.686, *T*_max_ = 0.854	*l* = −28→28
54661 measured reflections	

### Refinement

**Table:**

Refinement on *F*^2^	Secondary atom site location: difference Fourier map
Least-squares matrix: full	Hydrogen site location: inferred from neighbouring sites
*R*[*F*^2^ > 2σ(*F*^2^)] = 0.043	H-atom parameters constrained
*wR*(*F*^2^) = 0.125	*w* = 1/[σ^2^(*F*_o_^2^) + (0.0592*P*)^2^ + 0.6968*P*] where *P* = (*F*_o_^2^ + 2*F*_c_^2^)/3
*S* = 1.04	(Δ/σ)_max_ = 0.001
5600 reflections	Δρ_max_ = 0.29 e Å^−^^3^
420 parameters	Δρ_min_ = −0.20 e Å^−^^3^
0 restraints	Extinction correction: *SHELXL*, Fc^*^=kFc[1+0.001xFc^2^λ^3^/sin(2θ)]^-1/4^
Primary atom site location: structure-invariant direct methods	Extinction coefficient: 0.00104 (13)

### Special details

**Table:**

Geometry. All e.s.d.'s (except the e.s.d. in the dihedral angle between two l.s. planes) are estimated using the full covariance matrix. The cell e.s.d.'s are taken into account individually in the estimation of e.s.d.'s in distances, angles and torsion angles; correlations between e.s.d.'s in cell parameters are only used when they are defined by crystal symmetry. An approximate (isotropic) treatment of cell e.s.d.'s is used for estimating e.s.d.'s involving l.s. planes.
Refinement. Refinement of *F*^2^ against ALL reflections. The weighted *R*-factor *wR* and goodness of fit *S* are based on *F*^2^, conventional *R*-factors *R* are based on *F*, with *F* set to zero for negative *F*^2^. The threshold expression of *F*^2^ > σ(*F*^2^) is used only for calculating *R*-factors(gt) *etc*. and is not relevant to the choice of reflections for refinement. *R*-factors based on *F*^2^ are statistically about twice as large as those based on *F*, and *R*- factors based on ALL data will be even larger.

### Fractional atomic coordinates and isotropic or equivalent isotropic displacement parameters (Å^2^)

**Table:**

	*x*	*y*	*z*	*U*_iso_*/*U*_eq_	
F1	0.46395 (14)	0.11731 (9)	0.64805 (5)	0.0784 (4)	
F2	0.21171 (14)	0.44622 (9)	0.80466 (5)	0.0770 (4)	
O1	0.68735 (16)	0.23173 (9)	0.61437 (5)	0.0624 (4)	
O2	0.6461 (2)	0.10831 (9)	0.49564 (6)	0.0736 (4)	
O3	0.69794 (18)	0.56520 (9)	0.55258 (6)	0.0679 (4)	
O4	0.47768 (15)	0.39636 (9)	0.66732 (5)	0.0615 (4)	
O5	0.44069 (18)	0.59221 (9)	0.72372 (6)	0.0688 (4)	
O6	0.6323 (2)	0.16777 (10)	0.83123 (6)	0.0773 (4)	
C1	0.6664 (2)	0.25474 (12)	0.51551 (7)	0.0506 (4)	
C2	0.6927 (2)	0.18855 (13)	0.47863 (8)	0.0581 (5)	
C3	0.7666 (3)	0.20342 (15)	0.42802 (9)	0.0667 (6)	
H3	0.7845	0.1573	0.4029	0.080*	
C4	0.8120 (2)	0.28474 (15)	0.41561 (9)	0.0648 (6)	
H4	0.8624	0.2946	0.3815	0.078*	
C5	0.8356 (2)	0.43996 (15)	0.43901 (8)	0.0644 (5)	
H5	0.8902	0.4502	0.4059	0.077*	
C6	0.8059 (3)	0.50630 (15)	0.47336 (9)	0.0653 (6)	
H6	0.8397	0.5626	0.4642	0.078*	
C7	0.7247 (2)	0.49263 (13)	0.52286 (8)	0.0574 (5)	
C8	0.6796 (2)	0.41099 (12)	0.53769 (7)	0.0517 (4)	
H8	0.6274	0.4022	0.5715	0.062*	
C9	0.7109 (2)	0.34018 (12)	0.50253 (7)	0.0507 (4)	
C10	0.7868 (2)	0.35504 (14)	0.45149 (8)	0.0559 (5)	
C11	0.5998 (2)	0.23311 (12)	0.57105 (7)	0.0496 (4)	
C12	0.4255 (2)	0.21331 (12)	0.57099 (7)	0.0476 (4)	
C13	0.3632 (2)	0.15833 (13)	0.60998 (8)	0.0547 (5)	
C14	0.2027 (2)	0.13971 (14)	0.61020 (9)	0.0625 (5)	
H14	0.1650	0.1007	0.6371	0.075*	
C15	0.0981 (2)	0.17842 (15)	0.57095 (9)	0.0652 (5)	
H15	−0.0135	0.1668	0.5709	0.078*	
C16	0.1535 (2)	0.23433 (15)	0.53132 (8)	0.0643 (5)	
H16	0.0803	0.2612	0.5043	0.077*	
C17	0.3159 (2)	0.25097 (13)	0.53125 (8)	0.0558 (5)	
H17	0.3536	0.2887	0.5036	0.067*	
C18	0.6836 (4)	0.03622 (16)	0.46183 (11)	0.0900 (8)	
H18A	0.6311	0.0428	0.4237	0.108*	
H18B	0.6455	−0.0166	0.4790	0.108*	
H18C	0.8000	0.0329	0.4595	0.108*	
C19	0.6063 (3)	0.55635 (15)	0.60080 (9)	0.0675 (6)	
H19A	0.6662	0.5214	0.6296	0.081*	
H19B	0.5041	0.5282	0.5895	0.081*	
H19C	0.5859	0.6133	0.6164	0.081*	
C20	0.5035 (2)	0.45589 (13)	0.75890 (8)	0.0518 (4)	
C21	0.5205 (2)	0.54346 (13)	0.76503 (8)	0.0553 (5)	
C22	0.6132 (2)	0.57851 (14)	0.81180 (9)	0.0625 (5)	
H22	0.6273	0.6390	0.8152	0.075*	
C23	0.6827 (2)	0.52424 (15)	0.85231 (9)	0.0637 (6)	
H23	0.7432	0.5478	0.8841	0.076*	
C24	0.7374 (3)	0.37551 (17)	0.88765 (9)	0.0707 (6)	
H24	0.7963	0.3974	0.9203	0.085*	
C25	0.7240 (3)	0.28997 (18)	0.88072 (9)	0.0745 (6)	
H25	0.7740	0.2523	0.9082	0.089*	
C26	0.6366 (2)	0.25619 (14)	0.83326 (8)	0.0606 (5)	
C27	0.5636 (2)	0.30798 (13)	0.79316 (8)	0.0542 (5)	
H27	0.5044	0.2839	0.7613	0.065*	
C28	0.5765 (2)	0.39972 (13)	0.79942 (7)	0.0513 (4)	
C29	0.6658 (2)	0.43405 (14)	0.84741 (8)	0.0568 (5)	
C30	0.4085 (2)	0.41890 (12)	0.70827 (7)	0.0495 (4)	
C31	0.2319 (2)	0.40506 (12)	0.70876 (7)	0.0471 (4)	
C32	0.1502 (2)	0.37456 (13)	0.65940 (8)	0.0544 (5)	
H32	0.2086	0.3632	0.6273	0.065*	
C33	−0.0126 (2)	0.36050 (14)	0.65589 (9)	0.0603 (5)	
H33	−0.0657	0.3407	0.6215	0.072*	
C34	−0.0988 (2)	0.37524 (13)	0.70255 (9)	0.0617 (5)	
H34	−0.2113	0.3657	0.7003	0.074*	
C35	−0.0217 (2)	0.40370 (14)	0.75227 (9)	0.0618 (5)	
H35	−0.0802	0.4133	0.7846	0.074*	
C36	0.1410 (2)	0.41815 (13)	0.75471 (8)	0.0531 (5)	
C37	0.4591 (3)	0.68400 (14)	0.72680 (11)	0.0769 (7)	
H37A	0.4104	0.7057	0.7604	0.092*	
H37B	0.4061	0.7104	0.6928	0.092*	
H37C	0.5734	0.6986	0.7293	0.092*	
C38	0.5499 (3)	0.12902 (16)	0.78316 (10)	0.0816 (7)	
H38A	0.4366	0.1457	0.7814	0.098*	
H38B	0.5588	0.0663	0.7862	0.098*	
H38C	0.5977	0.1483	0.7488	0.098*	

### Atomic displacement parameters (Å^2^)

**Table:**

	*U*^11^	*U*^22^	*U*^33^	*U*^12^	*U*^13^	*U*^23^
F1	0.0612 (8)	0.0987 (9)	0.0760 (8)	0.0004 (6)	0.0083 (6)	0.0368 (7)
F2	0.0563 (7)	0.1236 (11)	0.0527 (6)	−0.0066 (7)	0.0160 (5)	−0.0252 (7)
O1	0.0554 (8)	0.0789 (9)	0.0521 (8)	−0.0110 (7)	−0.0022 (6)	0.0082 (6)
O2	0.0932 (12)	0.0620 (9)	0.0689 (9)	0.0009 (8)	0.0282 (8)	−0.0069 (7)
O3	0.0795 (10)	0.0635 (9)	0.0617 (8)	−0.0137 (7)	0.0117 (7)	0.0014 (7)
O4	0.0480 (8)	0.0895 (10)	0.0488 (7)	−0.0059 (7)	0.0148 (6)	−0.0126 (7)
O5	0.0769 (10)	0.0601 (8)	0.0692 (9)	−0.0042 (7)	0.0051 (8)	−0.0031 (7)
O6	0.0879 (11)	0.0740 (10)	0.0702 (10)	0.0165 (8)	0.0073 (8)	0.0055 (8)
C1	0.0420 (10)	0.0636 (11)	0.0470 (9)	0.0001 (8)	0.0084 (8)	0.0030 (8)
C2	0.0554 (12)	0.0648 (12)	0.0553 (11)	0.0028 (9)	0.0119 (9)	0.0020 (9)
C3	0.0667 (14)	0.0793 (15)	0.0562 (12)	0.0084 (11)	0.0190 (10)	−0.0045 (10)
C4	0.0549 (13)	0.0885 (16)	0.0532 (11)	0.0049 (11)	0.0184 (9)	0.0076 (10)
C5	0.0554 (12)	0.0864 (15)	0.0531 (11)	−0.0083 (11)	0.0147 (9)	0.0151 (11)
C6	0.0633 (13)	0.0736 (14)	0.0594 (12)	−0.0144 (11)	0.0085 (10)	0.0136 (10)
C7	0.0516 (12)	0.0679 (13)	0.0527 (11)	−0.0075 (9)	0.0022 (9)	0.0050 (9)
C8	0.0461 (11)	0.0634 (12)	0.0460 (10)	−0.0056 (8)	0.0061 (8)	0.0065 (8)
C9	0.0394 (10)	0.0665 (12)	0.0467 (10)	−0.0004 (8)	0.0064 (8)	0.0067 (8)
C10	0.0438 (11)	0.0763 (13)	0.0484 (10)	0.0002 (9)	0.0096 (8)	0.0076 (9)
C11	0.0476 (11)	0.0539 (10)	0.0476 (10)	−0.0005 (8)	0.0059 (8)	0.0008 (8)
C12	0.0458 (10)	0.0574 (11)	0.0406 (9)	−0.0007 (8)	0.0087 (7)	−0.0033 (7)
C13	0.0508 (11)	0.0656 (12)	0.0486 (10)	0.0017 (9)	0.0109 (8)	0.0035 (9)
C14	0.0533 (12)	0.0732 (13)	0.0632 (12)	−0.0033 (10)	0.0200 (10)	0.0037 (10)
C15	0.0461 (12)	0.0832 (15)	0.0676 (13)	−0.0043 (10)	0.0128 (10)	−0.0082 (11)
C16	0.0489 (12)	0.0879 (15)	0.0557 (11)	0.0035 (10)	0.0005 (9)	−0.0022 (10)
C17	0.0533 (12)	0.0708 (13)	0.0437 (10)	−0.0002 (9)	0.0064 (8)	0.0008 (8)
C18	0.122 (2)	0.0680 (15)	0.0831 (16)	0.0080 (14)	0.0304 (15)	−0.0129 (12)
C19	0.0698 (14)	0.0678 (13)	0.0658 (13)	−0.0097 (11)	0.0112 (11)	−0.0040 (10)
C20	0.0439 (10)	0.0637 (12)	0.0491 (10)	−0.0043 (8)	0.0132 (8)	−0.0070 (8)
C21	0.0484 (11)	0.0654 (12)	0.0536 (11)	−0.0058 (9)	0.0138 (9)	−0.0059 (9)
C22	0.0540 (12)	0.0706 (13)	0.0652 (13)	−0.0119 (10)	0.0198 (10)	−0.0188 (10)
C23	0.0454 (11)	0.0917 (16)	0.0553 (11)	−0.0079 (10)	0.0123 (9)	−0.0224 (11)
C24	0.0602 (14)	0.1036 (19)	0.0482 (11)	0.0051 (12)	0.0033 (9)	−0.0127 (11)
C25	0.0766 (16)	0.0989 (19)	0.0481 (11)	0.0189 (13)	0.0052 (10)	−0.0022 (11)
C26	0.0578 (13)	0.0745 (14)	0.0507 (11)	0.0087 (10)	0.0117 (9)	0.0025 (9)
C27	0.0458 (11)	0.0706 (12)	0.0469 (10)	0.0009 (9)	0.0092 (8)	−0.0057 (9)
C28	0.0395 (10)	0.0709 (12)	0.0450 (9)	−0.0026 (8)	0.0120 (8)	−0.0056 (9)
C29	0.0418 (11)	0.0839 (14)	0.0458 (10)	−0.0028 (9)	0.0104 (8)	−0.0137 (9)
C30	0.0464 (11)	0.0582 (11)	0.0450 (10)	−0.0026 (8)	0.0103 (8)	−0.0005 (8)
C31	0.0419 (10)	0.0558 (10)	0.0441 (9)	−0.0007 (8)	0.0068 (7)	0.0009 (8)
C32	0.0501 (11)	0.0691 (12)	0.0444 (10)	−0.0020 (9)	0.0063 (8)	−0.0001 (8)
C33	0.0456 (11)	0.0755 (13)	0.0588 (12)	−0.0062 (9)	−0.0023 (9)	−0.0012 (10)
C34	0.0403 (11)	0.0676 (13)	0.0775 (14)	−0.0030 (9)	0.0071 (10)	0.0038 (10)
C35	0.0468 (12)	0.0749 (13)	0.0661 (12)	0.0008 (9)	0.0205 (10)	−0.0022 (10)
C36	0.0469 (11)	0.0673 (12)	0.0458 (10)	0.0000 (9)	0.0085 (8)	−0.0054 (8)
C37	0.0886 (17)	0.0541 (12)	0.0903 (16)	−0.0022 (11)	0.0225 (13)	−0.0039 (11)
C38	0.0952 (19)	0.0676 (14)	0.0817 (16)	0.0102 (13)	0.0044 (14)	−0.0051 (12)

### Geometric parameters (Å, º)

**Table:**

F1—C13	1.349 (2)	C18—H18A	0.9800
F2—C36	1.356 (2)	C18—H18B	0.9800
O1—C11	1.217 (2)	C18—H18C	0.9800
O2—C2	1.376 (2)	C19—H19A	0.9800
O2—C18	1.426 (3)	C19—H19B	0.9800
O3—C7	1.357 (2)	C19—H19C	0.9800
O3—C19	1.427 (2)	C20—C21	1.376 (3)
O4—C30	1.216 (2)	C20—C28	1.404 (3)
O5—C21	1.372 (2)	C20—C30	1.504 (3)
O5—C37	1.437 (2)	C21—C22	1.414 (3)
O6—C26	1.376 (3)	C22—C23	1.375 (3)
O6—C38	1.421 (3)	C22—H22	0.9500
C1—C2	1.378 (3)	C23—C29	1.413 (3)
C1—C9	1.419 (3)	C23—H23	0.9500
C1—C11	1.504 (2)	C24—C25	1.344 (3)
C2—C3	1.407 (3)	C24—C29	1.419 (3)
C3—C4	1.358 (3)	C24—H24	0.9500
C3—H3	0.9500	C25—C26	1.398 (3)
C4—C10	1.410 (3)	C25—H25	0.9500
C4—H4	0.9500	C26—C27	1.356 (3)
C5—C6	1.348 (3)	C27—C28	1.437 (3)
C5—C10	1.419 (3)	C27—H27	0.9500
C5—H5	0.9500	C28—C29	1.418 (3)
C6—C7	1.413 (3)	C30—C31	1.493 (2)
C6—H6	0.9500	C31—C36	1.387 (2)
C7—C8	1.377 (3)	C31—C32	1.394 (2)
C8—C9	1.417 (3)	C32—C33	1.376 (3)
C8—H8	0.9500	C32—H32	0.9500
C9—C10	1.424 (2)	C33—C34	1.381 (3)
C11—C12	1.489 (3)	C33—H33	0.9500
C12—C13	1.387 (2)	C34—C35	1.374 (3)
C12—C17	1.394 (3)	C34—H34	0.9500
C13—C14	1.373 (3)	C35—C36	1.376 (3)
C14—C15	1.369 (3)	C35—H35	0.9500
C14—H14	0.9500	C37—H37A	0.9800
C15—C16	1.384 (3)	C37—H37B	0.9800
C15—H15	0.9500	C37—H37C	0.9800
C16—C17	1.383 (3)	C38—H38A	0.9800
C16—H16	0.9500	C38—H38B	0.9800
C17—H17	0.9500	C38—H38C	0.9800
			
C2—O2—C18	118.02 (17)	H19A—C19—H19C	109.5
C7—O3—C19	116.96 (16)	H19B—C19—H19C	109.5
C21—O5—C37	117.89 (17)	C21—C20—C28	120.44 (17)
C26—O6—C38	117.49 (17)	C21—C20—C30	120.50 (18)
C2—C1—C9	120.35 (17)	C28—C20—C30	119.05 (17)
C2—C1—C11	118.30 (17)	O5—C21—C20	115.47 (17)
C9—C1—C11	121.24 (16)	O5—C21—C22	123.80 (19)
O2—C2—C1	115.46 (17)	C20—C21—C22	120.7 (2)
O2—C2—C3	123.34 (19)	C23—C22—C21	119.4 (2)
C1—C2—C3	121.18 (19)	C23—C22—H22	120.3
C4—C3—C2	118.9 (2)	C21—C22—H22	120.3
C4—C3—H3	120.5	C22—C23—C29	121.13 (19)
C2—C3—H3	120.5	C22—C23—H23	119.4
C3—C4—C10	122.35 (18)	C29—C23—H23	119.4
C3—C4—H4	118.8	C25—C24—C29	121.7 (2)
C10—C4—H4	118.8	C25—C24—H24	119.1
C6—C5—C10	121.17 (18)	C29—C24—H24	119.1
C6—C5—H5	119.4	C24—C25—C26	120.3 (2)
C10—C5—H5	119.4	C24—C25—H25	119.9
C5—C6—C7	120.4 (2)	C26—C25—H25	119.9
C5—C6—H6	119.8	C27—C26—O6	124.01 (19)
C7—C6—H6	119.8	C27—C26—C25	121.5 (2)
O3—C7—C8	125.14 (18)	O6—C26—C25	114.49 (19)
O3—C7—C6	114.37 (18)	C26—C27—C28	119.36 (18)
C8—C7—C6	120.49 (19)	C26—C27—H27	120.3
C7—C8—C9	120.04 (17)	C28—C27—H27	120.3
C7—C8—H8	120.0	C20—C28—C29	119.44 (19)
C9—C8—H8	120.0	C20—C28—C27	121.40 (17)
C8—C9—C1	122.51 (16)	C29—C28—C27	119.17 (18)
C8—C9—C10	119.07 (17)	C23—C29—C28	118.88 (19)
C1—C9—C10	118.41 (18)	C23—C29—C24	123.12 (19)
C4—C10—C5	122.54 (18)	C28—C29—C24	118.0 (2)
C4—C10—C9	118.76 (18)	O4—C30—C31	119.49 (16)
C5—C10—C9	118.70 (19)	O4—C30—C20	119.50 (16)
O1—C11—C12	121.72 (16)	C31—C30—C20	120.92 (14)
O1—C11—C1	120.24 (16)	C36—C31—C32	116.43 (16)
C12—C11—C1	118.04 (15)	C36—C31—C30	125.70 (16)
C13—C12—C17	116.63 (17)	C32—C31—C30	117.85 (15)
C13—C12—C11	122.78 (16)	C33—C32—C31	121.83 (17)
C17—C12—C11	120.59 (16)	C33—C32—H32	119.1
F1—C13—C14	117.43 (17)	C31—C32—H32	119.1
F1—C13—C12	119.34 (17)	C32—C33—C34	119.77 (19)
C14—C13—C12	123.14 (18)	C32—C33—H33	120.1
C15—C14—C13	118.77 (19)	C34—C33—H33	120.1
C15—C14—H14	120.6	C35—C34—C33	120.02 (19)
C13—C14—H14	120.6	C35—C34—H34	120.0
C14—C15—C16	120.52 (19)	C33—C34—H34	120.0
C14—C15—H15	119.7	C34—C35—C36	119.28 (18)
C16—C15—H15	119.7	C34—C35—H35	120.4
C17—C16—C15	119.7 (2)	C36—C35—H35	120.4
C17—C16—H16	120.1	F2—C36—C35	117.15 (16)
C15—C16—H16	120.1	F2—C36—C31	120.21 (16)
C16—C17—C12	121.20 (18)	C35—C36—C31	122.65 (18)
C16—C17—H17	119.4	O5—C37—H37A	109.5
C12—C17—H17	119.4	O5—C37—H37B	109.5
O2—C18—H18A	109.5	H37A—C37—H37B	109.5
O2—C18—H18B	109.5	O5—C37—H37C	109.5
H18A—C18—H18B	109.5	H37A—C37—H37C	109.5
O2—C18—H18C	109.5	H37B—C37—H37C	109.5
H18A—C18—H18C	109.5	O6—C38—H38A	109.5
H18B—C18—H18C	109.5	O6—C38—H38B	109.5
O3—C19—H19A	109.5	H38A—C38—H38B	109.5
O3—C19—H19B	109.5	O6—C38—H38C	109.5
H19A—C19—H19B	109.5	H38A—C38—H38C	109.5
O3—C19—H19C	109.5	H38B—C38—H38C	109.5
			
C18—O2—C2—C1	−174.3 (2)	C37—O5—C21—C20	−177.68 (17)
C18—O2—C2—C3	3.9 (3)	C37—O5—C21—C22	3.2 (3)
C9—C1—C2—O2	179.53 (17)	C28—C20—C21—O5	−178.40 (16)
C11—C1—C2—O2	3.4 (3)	C30—C20—C21—O5	2.6 (2)
C9—C1—C2—C3	1.2 (3)	C28—C20—C21—C22	0.7 (3)
C11—C1—C2—C3	−174.89 (18)	C30—C20—C21—C22	−178.31 (16)
O2—C2—C3—C4	−178.3 (2)	O5—C21—C22—C23	177.09 (17)
C1—C2—C3—C4	−0.2 (3)	C20—C21—C22—C23	−2.0 (3)
C2—C3—C4—C10	−0.4 (3)	C21—C22—C23—C29	1.3 (3)
C10—C5—C6—C7	−0.1 (3)	C29—C24—C25—C26	−0.6 (3)
C19—O3—C7—C8	−4.5 (3)	C38—O6—C26—C27	1.9 (3)
C19—O3—C7—C6	175.66 (18)	C38—O6—C26—C25	−178.0 (2)
C5—C6—C7—O3	−177.94 (19)	C24—C25—C26—C27	0.0 (3)
C5—C6—C7—C8	2.2 (3)	C24—C25—C26—O6	180.0 (2)
O3—C7—C8—C9	178.56 (18)	O6—C26—C27—C28	−179.71 (17)
C6—C7—C8—C9	−1.6 (3)	C25—C26—C27—C28	0.2 (3)
C7—C8—C9—C1	−179.65 (18)	C21—C20—C28—C29	1.2 (3)
C7—C8—C9—C10	−1.0 (3)	C30—C20—C28—C29	−179.77 (15)
C2—C1—C9—C8	176.93 (18)	C21—C20—C28—C27	−178.46 (16)
C11—C1—C9—C8	−7.1 (3)	C30—C20—C28—C27	0.6 (2)
C2—C1—C9—C10	−1.7 (3)	C26—C27—C28—C20	179.75 (17)
C11—C1—C9—C10	174.33 (17)	C26—C27—C28—C29	0.1 (3)
C3—C4—C10—C5	179.3 (2)	C22—C23—C29—C28	0.6 (3)
C3—C4—C10—C9	−0.1 (3)	C22—C23—C29—C24	178.98 (18)
C6—C5—C10—C4	178.1 (2)	C20—C28—C29—C23	−1.8 (3)
C6—C5—C10—C9	−2.5 (3)	C27—C28—C29—C23	177.82 (16)
C8—C9—C10—C4	−177.56 (18)	C20—C28—C29—C24	179.71 (17)
C1—C9—C10—C4	1.1 (3)	C27—C28—C29—C24	−0.7 (3)
C8—C9—C10—C5	3.1 (3)	C25—C24—C29—C23	−177.5 (2)
C1—C9—C10—C5	−178.28 (18)	C25—C24—C29—C28	0.9 (3)
C2—C1—C11—O1	102.8 (2)	C21—C20—C30—O4	95.2 (2)
C9—C1—C11—O1	−73.3 (2)	C28—C20—C30—O4	−83.9 (2)
C2—C1—C11—C12	−76.9 (2)	C21—C20—C30—C31	−88.1 (2)
C9—C1—C11—C12	107.1 (2)	C28—C20—C30—C31	92.8 (2)
O1—C11—C12—C13	−29.1 (3)	O4—C30—C31—C36	172.05 (19)
C1—C11—C12—C13	150.62 (18)	C20—C30—C31—C36	−4.6 (3)
O1—C11—C12—C17	150.16 (19)	O4—C30—C31—C32	−6.5 (3)
C1—C11—C12—C17	−30.2 (3)	C20—C30—C31—C32	176.86 (17)
C17—C12—C13—F1	177.37 (17)	C36—C31—C32—C33	1.8 (3)
C11—C12—C13—F1	−3.4 (3)	C30—C31—C32—C33	−179.56 (18)
C17—C12—C13—C14	0.8 (3)	C31—C32—C33—C34	−1.2 (3)
C11—C12—C13—C14	−179.98 (18)	C32—C33—C34—C35	−0.1 (3)
F1—C13—C14—C15	−178.09 (18)	C33—C34—C35—C36	0.7 (3)
C12—C13—C14—C15	−1.4 (3)	C34—C35—C36—F2	179.97 (19)
C13—C14—C15—C16	0.9 (3)	C34—C35—C36—C31	−0.1 (3)
C14—C15—C16—C17	0.2 (3)	C32—C31—C36—F2	178.79 (17)
C15—C16—C17—C12	−0.9 (3)	C30—C31—C36—F2	0.3 (3)
C13—C12—C17—C16	0.4 (3)	C32—C31—C36—C35	−1.2 (3)
C11—C12—C17—C16	−178.86 (18)	C30—C31—C36—C35	−179.69 (19)
