# Supplementary material for: Crystal structure of 1-(2-fluoro­benzo­yl)-2,7-di­meth­oxy­naphthalene
Source: Acta Crystallogr Sect E Struct Rep Online. 2014 Oct 4;70(Pt 11):278–80. doi: 10.1107/S1600536814020807 (PMC4257270; doi:10.1107/S1600536814020807)
Supplement: Supplementary file 3 [file e-70-00278-Isup3.pdf]

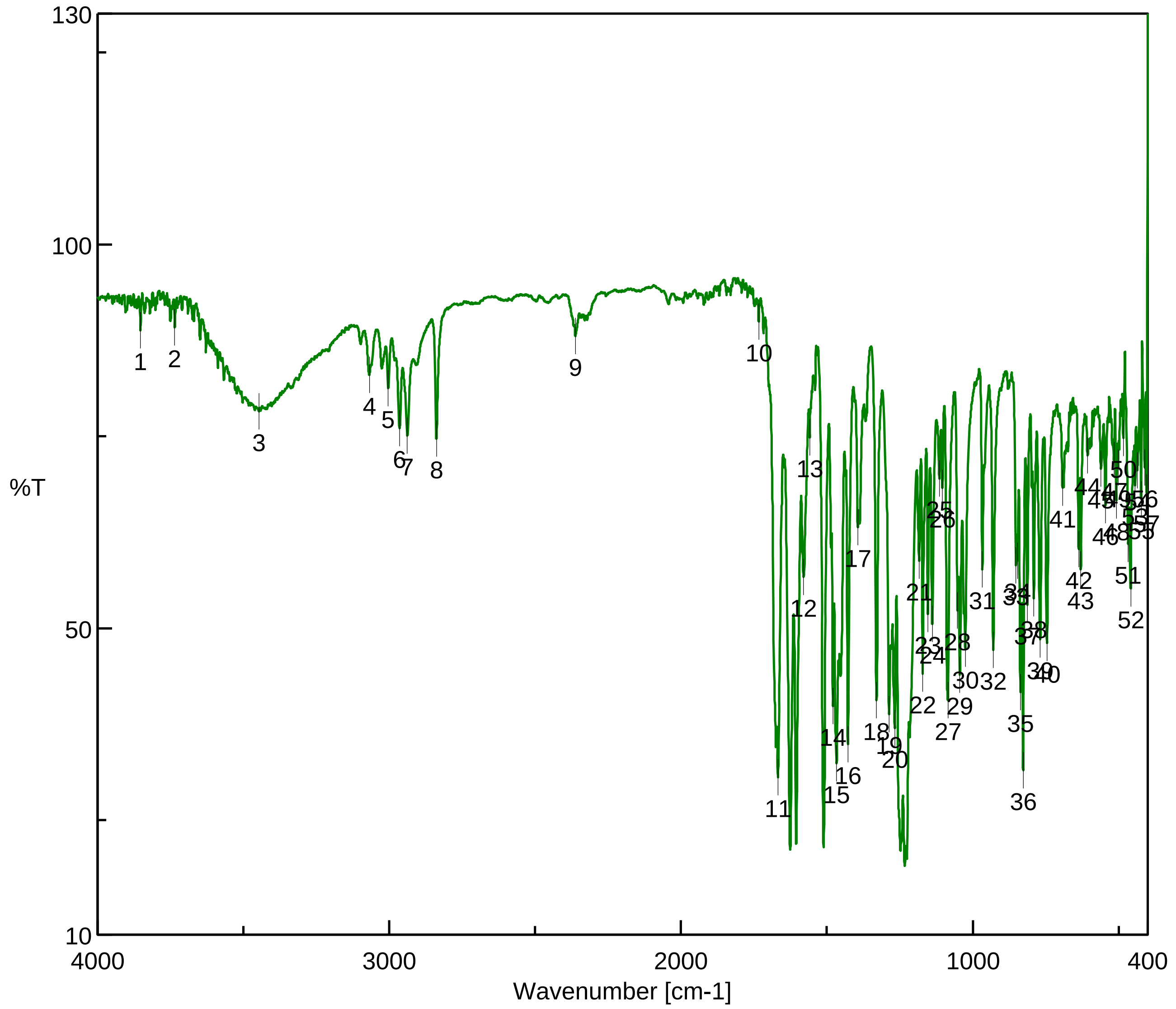

ピーク検出結果

No.	位置	強度	No.	位置	強度
1	3853.08	88.7285	2	3735.44	89.0858
3	3446.65	78.1913	4	3068.67	82.9109
5	3003.59	81.2183	6	2965.02	76.0151
7	2938.02	75.0405	8	2838.22	74.6371
9	2362.37	88.0194	10	1733.69	89.8816
11	1667.64	30.5025	12	1579.9	56.5937
13	1558.68	74.7812	14	1479.13	39.7596
15	1466.12	32.3156	16	1428.03	34.8049
17	1393.8	63.0846	18	1330.16	40.5196
19	1286.77	38.753	20	1267	36.9136
21	1183.6	58.7581	22	1171.54	44.0134
23	1153.71	51.79	24	1138.76	50.4673
25	1114.17	69.4648	26	1104.05	68.2122
27	1085.24	40.5365	28	1051.98	52.2001
29	1043.78	43.8477	30	1025.46	47.2428
31	967.126	57.5632	32	930.003	47.1389
33	851.418	58.0892	34	846.597	58.6877
35	835.508	41.6084	36	827.312	31.4356
37	812.849	52.9857	38	790.671	53.8572
39	768.012	48.449	40	745.352	48.0065
41	692.32	68.2094	42	636.876	60.2656
43	629.162	57.5897	44	606.021	72.4823
45	560.22	70.6791	46	545.274	65.9726
47	515.865	71.8561	48	507.669	66.6009
49	501.884	70.8902	50	484.045	74.6917
51	466.689	60.9376	52	458.493	55.1006
53	443.065	68.5257	54	436.316	70.4862
55	422.334	66.7313	56	410.281	70.8399
57	404.014	67.6101			

[コメント情報]

試料名	
コメント	
測定者	
所属	
会社	東京農工大学

[測定情報]

機種名	FT/IR-4100typeA
シリアル番号	B041461016
光源	標準光源
検出器	TGS
積算回数	16
分解	2 cm−1
ゼロフィリング	On
アポダイゼーション	Cosine
ゲイン	Auto (8)
アパーチャー	Auto (5 mm)
スキャンスピード	Auto (2 mm/sec)
フィルタ	Auto (30000 Hz)
