# Supplementary material for: Crystal structure of 1-(2-fluoro­benzo­yl)-2,7-di­meth­oxy­naphthalene
Source: Acta Crystallogr Sect E Struct Rep Online. 2014 Oct 4;70(Pt 11):278–80. doi: 10.1107/S1600536814020807 (PMC4257270; doi:10.1107/S1600536814020807)
Supplement: Supplementary file 5 [file e-70-00278-Isup5.pdf]

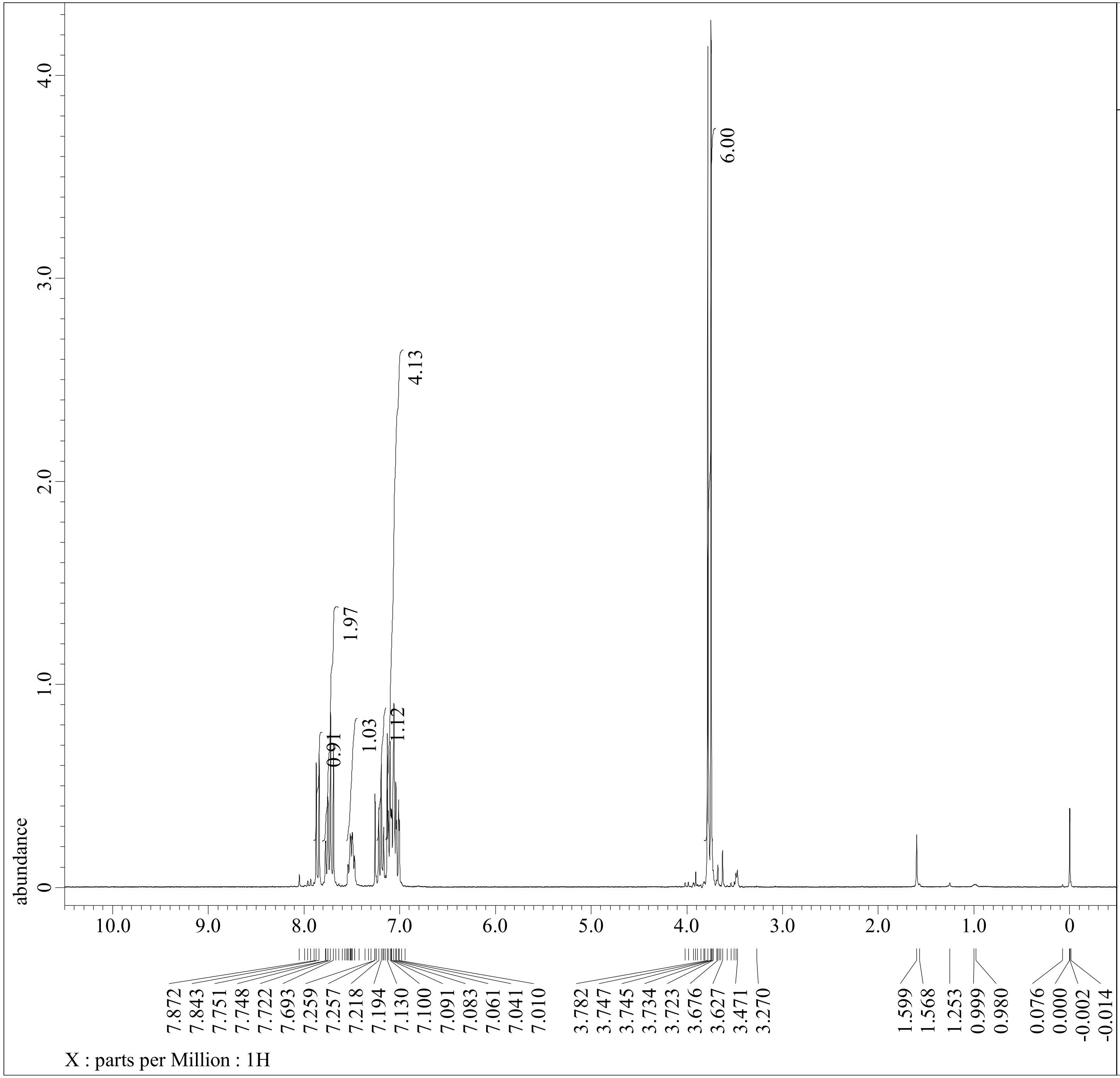

Filename	=	20140207-oF-ok-3.jdf
Author	=	delta
Experiment	=	single_pulse.ex2
Sample_Id	=	S#612288
Solvent	=	CHLOROFORM-D
Creation_Time	=	7-FEB-2014 16:42:15
Revision_Time	=	10-FEB-2014 13:38:09
Current_Time	=	2-SEP-2014 16:27:57
Comment	=	single_pulse
Data_Format	=	1D COMPLEX
Dim_Size	=	13107
Dim_Title	=	1H
Dim_Units	=	[ppm]
Dimensions	=	X
Site	=	ECX 300
Spectrometer	=	JNM-ECX300
Field_Strength	=	7.0586013[T] (300[MHz])
X_Acq_Duration	=	2.90717696[s]
X_Domain	=	1H
X_Freq	=	300.52965592[MHz]
X_Offset	=	5[ppm]
X_Points	=	16384
X_Prescans	=	1
X_Resolution	=	0.34397631[Hz]
X_Sweep	=	5.63570784[kHz]
Irr_Domain	=	1H
Irr_Freq	=	300.52965592[MHz]
Irr_Offset	=	5[ppm]
Tri_Domain	=	1H
Tri_Freq	=	300.52965592[MHz]
Tri_Offset	=	5[ppm]
Clipped	=	FALSE
Scans	=	4
Total_Scans	=	4
Relaxation_Delay	=	5[s]
Recvr_Gain	=	38
Temp_Get	=	17.1[dC]
X_90_Width	=	17.75[us]
X_Acq_Time	=	2.90717696[s]
X_Angle	=	45[deg]
X_Atn	=	7.06[dB]
X_Pulse	=	8.875[us]
Irr_Mode	=	Off
Tri_Mode	=	Off
Dante_Presat	=	FALSE
Initial_Wait	=	1[s]
Repetition_Time	=	7.90717696[s]
