# Supplementary material for: Crystal structure of 1-(2-fluoro­benzo­yl)-2,7-di­meth­oxy­naphthalene
Source: Acta Crystallogr Sect E Struct Rep Online. 2014 Oct 4;70(Pt 11):278–80. doi: 10.1107/S1600536814020807 (PMC4257270; doi:10.1107/S1600536814020807)
Supplement: Supplementary file 6 [file e-70-00278-Isup6.pdf]

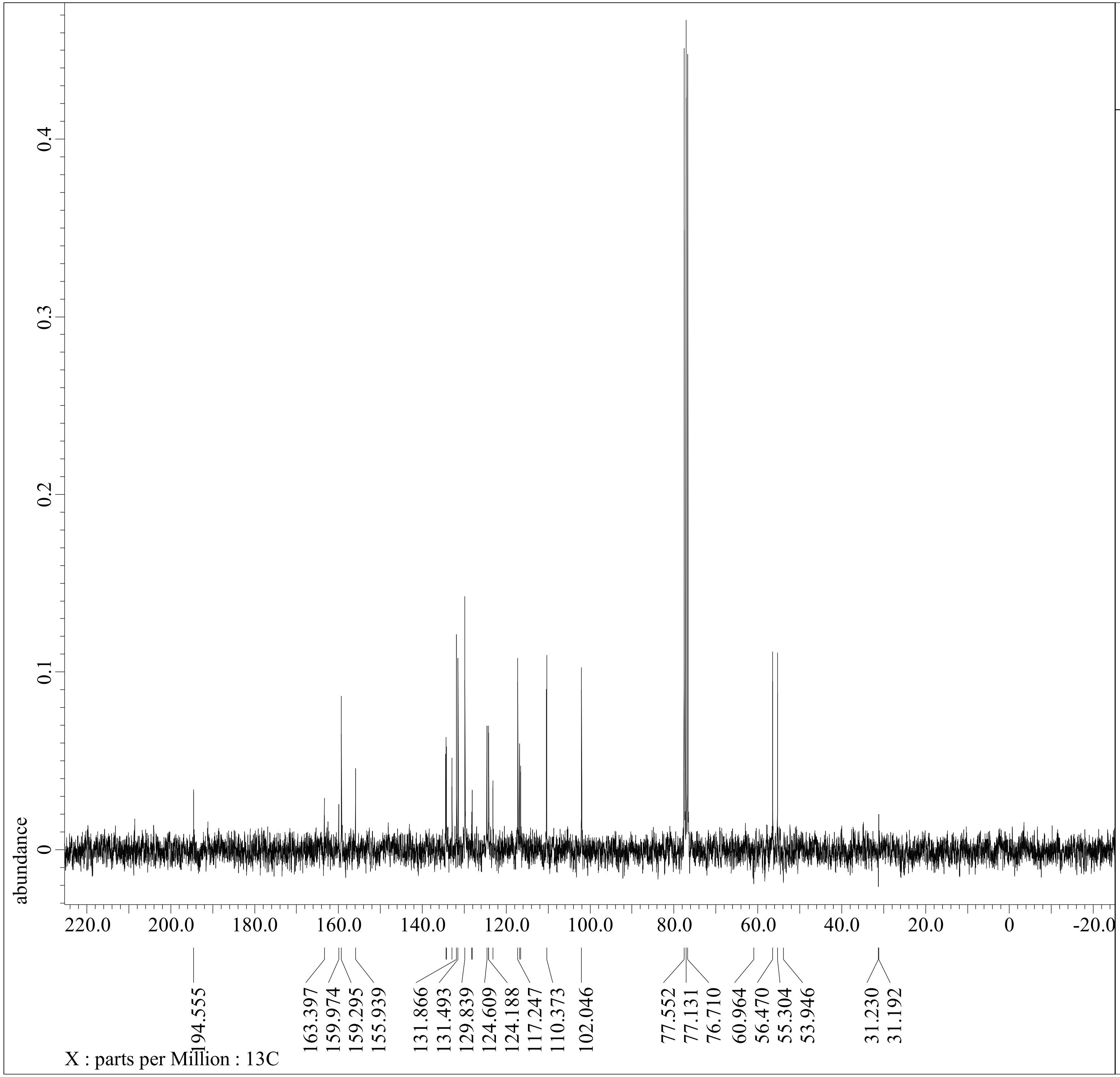

JEOL RESONANCE

```
Filename          = 140801_mono-o-F-ok-1.jdf
Author            = delta
Experiment        = single_pulse_dec
Sample_Id         = S#632103
Solvent           = CHLOROFORM-D
Creation_Time     =  1-AUG-2014 16:58:35
Revision_Time     =  5-AUG-2014 17:04:11
Current_Time      =  3-SEP-2014 10:43:21

Comment           = single pulse decoupled g
Data_Format       = 1D COMPLEX
Dim_Size          = 26214
Dim_Title         = 13C
Dim_Units         = [ppm]
Dimensions        = X
Site              = ECX 300
Spectrometer      = JNM-ECX300

Field_Strength    = 7.0586013[T] (300[MHz])
X_Acq_Duration    = 1.38412032[s]
X_Domain          = 13C
X_Freq            = 75.56823426[MHz]
X_Offset          = 100[ppm]
X_Points          = 32768
X_Prescans        = 4
X_Resolution      = 0.72248054[Hz]
X_Sweep           = 23.67424242[kHz]
Irr_Domain        = 1H
Irr_Freq          = 300.52965592[MHz]
Irr_Offset        = 5[ppm]
Clipped           = FALSE
Scans             = 88
Total_Scans       = 88

Relaxation_Delay  = 2[s]
Recvr_Gain        = 60
Temp_Get          = 24.2[dC]
X_90_Width        = 9.5[us]
X_Acq_Time        = 1.38412032[s]
X_Angle           = 30[deg]
X_Atn             = 5.3[dB]
X_Pulse           = 3.16666667[us]
Irr_Atn_Dec       = 21.35[dB]
Irr_Atn_Noe       = 21.35[dB]
Irr_Noise         = WALTZ
Decoupling        = TRUE
Initial_Wait      = 1[s]
Noe               = TRUE
Noe_Time          = 2[s]
Repetition_Time   = 3.38412032[s]
```
